# Combination of Aptamer Amplifier and Antigen-Binding Fragment Probe as a Novel Strategy to Improve Detection Limit of Silicon Nanowire Field-Effect Transistor Immunosensors

**DOI:** 10.3390/s21020650

**Published:** 2021-01-19

**Authors:** Cao-An Vu, Pin-Hsien Pan, Yuh-Shyong Yang, Hardy Wai-Hong Chan, Yoichi Kumada, Wen-Yih Chen

**Affiliations:** 1 Department of Chemical and Materials Engineering, National Central University, Jhongli 320, Taiwan; vucaoan@gmail.com (C.-A.V.); mm85322@gmail.com (P.-H.P.); 2 Institute of Biological Science and Technology, National Chiao Tung University, Hsinchu 300, Taiwan; ysyang@cc.nctu.edu.tw; 3 Helios Bioelectronics Inc., Hsinchu 30261, Taiwan; HWChan@rocketmail.com; 4 Department of Functional Chemistry and Engineering, Kyoto Institute of Technology, 1 Matsugasaki-Hashikami-Cho, Sakyo-ku, Kyoto 606-0951, Japan; kumada@kit.ac.jp

**Keywords:** silicon nanowire field-effect transistor biosensor, aptamer, Fab, ELISA, mixed self-assembled monolayer, signal enhancement

## Abstract

Detecting proteins at low concentrations in high-ionic-strength conditions by silicon nanowire field-effect transistors (SiNWFETs) is severely hindered due to the weakened signal, primarily caused by screening effects. In this study, aptamer as a signal amplifier, which has already been reported by our group, is integrated into SiNWFET immunosensors employing antigen-binding fragments (Fab) as the receptors to improve its detection limit for the first time. The Fab-SiNWFET immunosensors were developed by immobilizing Fab onto Si surfaces modified with either 3-aminopropyltriethoxysilane (APTES) and glutaraldehyde (GA) (Fab/APTES-SiNWFETs), or mixed self-assembled monolayers (mSAMs) of polyethylene glycol (PEG) and GA (Fab/PEG-SiNWFETs), to detect the rabbit IgG at different concentrations in a high-ionic-strength environment (150 mM Bis-Tris Propane) followed by incubation with R18, an aptamer which can specifically target rabbit IgG, for signal enhancement. Empirical results revealed that the signal produced by the sensors with Fab probes was greatly enhanced compared to the ones with whole antibody (Wab) after detecting similar concentrations of rabbit IgG. The Fab/PEG-SiNWFET immunosensors exhibited an especially improved limit of detection to determine the IgG level down to 1 pg/mL, which has not been achieved by the Wab/PEG-SiNWFET immunosensors.

## 1. Introduction

Silicon nanowire field-effect transistor (SiNWFET) biosensors have been demonstrated as ultra-sensitive devices which can provide the real-time and label-free detection of numerous targets [1], especially proteins, by so-called immunosensors [2]. Furthermore, they are low-cost and have the potential to be commercialized for large-scale applications because of the possibility of mass-production by the semiconductor industry of SiNWFET [3]. However, detecting proteins at low concentrations by SiNWFET immunosensors is severely hindered due to the low signal-to-noise ratio from intrinsic properties of the devices [4] and weakened signal in high-ionic-strength conditions (screening effect, also known as Debye length) [5,6]. Consequently, exploiting SiNWFET immunosensors for sensing applications is limited by deteriorated sensitivity, especially in disease diagnosis with proteins at ultra-low concentrations. Boosting and optimizing the sensitivity of SiNWFET immunosensors has, therefore, become inevitable for practical trials in diagnosis applications.

The antibody is the most popular receptor employed in SiNWFET immunosensors [1], a kind of SiNWFET biosensor where the signal from immunoassays is recorded and modulated by SiNWFET transducers [2] because of its simplicity, feasibility for immobilization on a wide range of diverse surfaces and its ability to provide fast detection via high-specificity and -affinity binding with the target molecules [7]. Nevertheless, the bulky size of antibodies (roughly 14.5 × 8.5 × 4.0 nm for the IgG isotype [8]) may exceed the Debye length of high-ionic-strength environments (approximately 0.7 nm [5,9,10]) and let the captured antigen move far from the detectable region of SiNWFET transducers [11]. As a result, the detected signal is weakened and the sensitivity of the SiNWFET immunosensors is deteriorated in physiological conditions [12], posing an obstacle to the clinical diagnosis applications of SiNWFET immunosensors. Antigen-binding fragments (Fab) have been evidenced as a replacement of the antibody in FET immunosensors to improve their sensitivity and limit of detection (LOD) [10,13]. Not only do Fabs possess the aforementioned advantages of antibody, but they also have smaller dimensions, allowing Fabs to be immobilized on the nanowire surface at a higher density than the antibody, and Fab-target binding events occur in areas closer to the sensing channels than that of the antibody-antigen [7]. Consequently, the electrical signal recorded by the transducers is enhanced and the quantification range of the immunosensors is widened.

An amplifier is indispensable for SiNWFET biosensors, helping them to eliminate interferences from intrinsic properties of the transducers and physiological environments to boost their signal-to-noise ratio [4,14,15,16]. Recently, aptamer has emerged as a promising candidate for this duty, since it has been demonstrated as a bio-amplifier for SiNWFET immmunosensors by stabilizing and amplifying the signal recorded from both direct and sandwich assays [17]. Furthermore, the amplified signal by aptamer is also applicable for quantifying proteins in high-ionic-strength conditions [18]. Surface modification methods can also contribute to optimizing the sensitivity of the SiNWFET immunosensors. On the one hand, polyethylene glycol (PEG) is accredited with ability to increase Debye length and expand the detectable region of FET-based biosensors by substantially changing the dielectric properties of high-ionic-strength solution [6,19,20,21,22,23]. On the other hand, it is very popular in fouling-resistance applications because the PEG layer can create steric repulsion and form a hydrated layer near the coated surface, acting as a barrier to the non-specific adsorption of unwanted species [24,25]. In addition to that, the spacer effect from the flexible chain of PEG and its hydrophylic environment can retain the bioactivities of recognition factors and, correspondingly, contribute to the sensitivity of biosensors [25,26]. Mixed self-assembled monolayers (mSAMs) are a confirmed approach to the anti-fouling function of biosensors [27,28,29], especially for embedding PEG onto FET-sensing channels, with recent publications [20,21,22,23].

In this study, we immobilized Fabs onto SiNWFET surfaces, modified with mSAMs of silane-PEGs (silane-PEG-NH_2_ and silane-PEG-OH) to detect rabbit IgG. R18, an RNA aptamer which can specifically bind with rabbit IgG [30], is employed as the amplifier to stabilize and enhance the signal produced from protein detections in high-ionic-strength conditions for the as-designed sensors. Theoretically, assembling SiNWFET, Fab, mixed-SAM, and aptamer can release a novel generation of ultrasensitive FET-based biosensors, overcoming the limitations of Debye length and interference from high-ionic-strength environments. Empirical data reveal that, for both of the sensors manufactured by modifying SiNWFET surfaces with either APTES and glutaraldehyde (GA) or PEG-mSAM and GA, the amplified signal generated by the ones with Fab probes was greater than the ones with whole antibody (Wab) probes after detecting similar concentrations of rabbit IgG. Moreover, the Fab/PEG-SiNWFET immunosensors exhibited an improved limit of detection to determine the IgG level down to 1 pg/mL, which was not achieved by the Wab/PEG-SiNWFET immunosensors. The FET results were supported by the successful surface modification and antibody-antigen detections verified from the indirect ELISA and Langmuir adsorption model, which also evidenced that (1) the signal changes recorded by the fabricated FETs were induced from their specific binding and (2) the synergic amplification effect of integrating both Fab and aptamer into the SiNWFET immunosensors were from the compact structure of the Fab.

## 2. Materials and Methods

### 2.1. Materials

3-Aminopropyltriethoxysilane (APTES), GA, Bis-Tris Propane (BTP), Sodium Cyanoborohydride (NaBH_3_CN), Tris(hydroxymethyl)aminomethane (Tris), and Hydrochloric Acid (HCl) were delivered by Sigma-Aldrich. 3,3′,5,5′-Tetramethylbenzidine (TMB), acetone, and ethanol (99.9%) was ordered from Thermo Fisher Scientific, while the components of PEG-mSAMs (silane-PEG-NH_2_, 1K and silane-PEG-OH, 1K) were from Biochempeg Scientific Inc. All the antibodies, including rabbit immunoglobulin G (IgG), donkey anti-goat antibody conjugated with horseradish peroxide (HRP), goat anti-rabbit IgG antibody (Wab), and its Fab, were supplied by Abcam plc. (UK), whereas R18 aptamer specifying rabbit IgG (74-mer RNA sequence: 5′-GGGAG AAUUC CGACC AGAAG UUCGA UACGC CGUGG GGUGA CGUUG GCUAC CCUUU CCUCU CUCCU CCUUC UUCU-3′ [28]) was synthesized by MDBio, Inc. Meanwhile, 10 mM Tris, 10 mM and 150 mM BTP buffer were all prepared in deionized water and the pH was adjusted to 7.4 by HCl. PBST solution was made by mixing 0.2% Tween 20 (Polysorbate 20 from Showa corporation, Japan) in 1 × PBS (phosphate buffer saline from Genestar Biotech Ltd., Taiwan). The other chemicals for this research were reagent grade.

### 2.2. Apparatuses and Characteristics of SiNWFET

The electrical response of biosensing performance by SiNWFET immunosensors, which were fabricated from n-type devices provided by Episil Technologies Inc. (Hsinchu, Taiwan) and have been used in our recent publications [17,18,31,32,33], were collected by Keithley 2636 Dual-Channel System Source Meter Instrument and a probe station with a chamber (Everbeing).

### 2.3. Fabrication of SiNWFET Immunosensors

There were four kinds of SiNWFET immunosensor used in this research for biosensing and signal enhancement. All of them were manufactured by initially washing SiNWFET devices with acetone, ethanol, and deionized (DI) water to remove impurities and treating them with oxygen plasma for 5 min to prepare for surface modification.

#### 2.3.1. Fabrication of Wab/APTES-SiNWFET and Fab/APTES-SiNWFET Immunosensors

**Figure 1:**
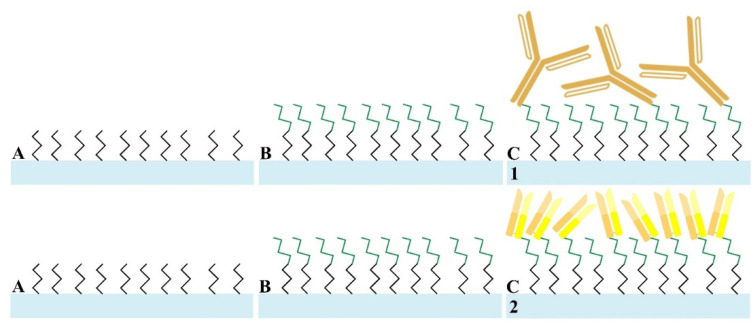
Depiction of the fabrication of Wab/APTES-SiNWFET (sample 1) and Fab/APTES-SiNWFET (sample 2) immunosensors in this study. (**A**) The SiNW channels (light blue bar) were modified with APTES (black short zigzag shapes) and (**B**) GA (green zigzag shapes) before (**C**) immobilizing either the Wab (bronze Y shapes in sample 1) or the Fab (yellow-bronze bars in sample 2).

The Wab/APTES-SiNWFETs (sample 1 in Figure 1) and Fab/APTES-SiNWFETs (sample 2 in Figure 1) were produced by initially shaking treated SiNWFETs in a 2%-APTES solution (with 99.9% ethanol as the solvent), followed by washing with 95% ethanol, and heated at 120 °C for 10 min (Figure 1A). They were then functionalized with 2.5% GA in 10 mM BTP for 60 min and washed by DI-water (Figure 1B) to immobilize the bio-probes (Wab or Fab).

The bio-probes (Wab or Fab) were immobilized onto the APTES/GA-modified devices by incubating them in 10 mM-BTP solution containing 0.4% NaBH_3_CN and either 1 μg/mL Wab or 1 μg/mL Fab at 4 °C overnight. The next day, they were all washed by DI-water in order to remove non-specific binding of the biomolecules on the nanowire surface before being incubated with 10 mM Tris-HCl containing 0.4% NaBH_3_CN as a blocking buffer in 30 min. These bio-chips (called Wab/APTES-SiNWFETs in sample 1 and Fab/APTES-SiNWFETs in sample 2 of Figure 1C) were finally purified by DI-water and desiccated by nitrogen to prepare for the biosensing performance. The manufacturing processes of two-type FET immunosensors by modifying SiNW surfaces with APTES are illustrated in Figure 1.

#### 2.3.2. Fabrication of Wab/PEG-SiNWFET and Fab/PEG-SiNWFET Immunosensors

**Figure 2:**
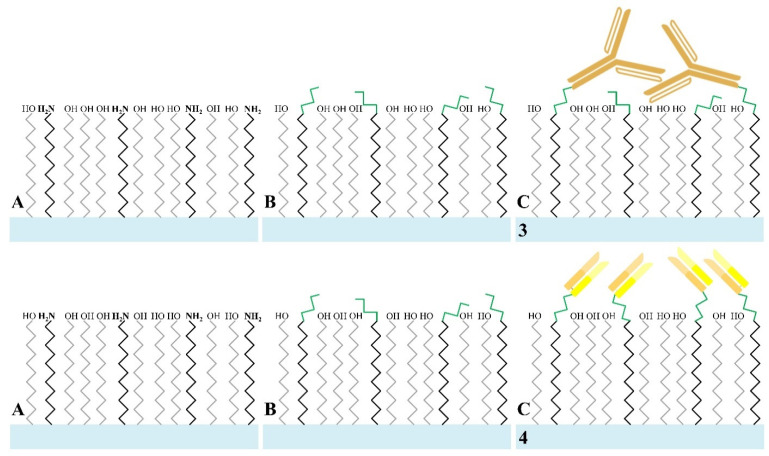
Depiction of the fabrication of Wab/PEG-SiNWFET (sample 3) and Fab/PEG-SiNWFET (sample 4) immunosensors in this study. (**A**) The SiNW channels (light blue bar) were modified with PEG-mSAMs (silane-PEG-NH_2_: black long zigzag shapes, silane-PEG-OH: grey long zigzag shapes) and (**B**) GA (green zigzag shapes) before (**C**) either the Wab (bronze Y shapes in sample 3) or the Fab (yellow-bronze bars in sample 4) were immobilized.

The Wab/PEG-SiNWFETs (sample 3 in Figure 2) and Fab/PEG-SiNWFETs (sample 4 in Figure 2) were produced by initially shaking treated SiNWFETs in an mSAM solution composed of 20 mg silane-PEG-OH and 2 mg silane-PEG-NH_2_ in 2 mL 99.9% ethanol for 30 min on a platform rocker at ambient temperature, followed by washing with 95% ethanol, and heated at 120 °C for 10 min (Figure 2A). They were then functionalized with 2.5% GA in 10 mM BTP for 60 min and washed by DI-water (Figure 2B) to immobilize the bio-probes (Wab or Fab).

The bio-probes (Wab or Fab) were immobilized onto the PEG/GA-modified devices by incubating them in 10 mM-BTP solution containing 0.4% NaBH_3_CN and either 1 μg/mL Wab or 1 μg/mL Fab at 4 °C overnight. The next day, they were initially washed by DI-water in order to remove non-specific binding of the biomolecules on the nanowire surface, before being incubated with 10 mM Tris-HCl containing 0.4% NaBH_3_CN as a blocking buffer in 30 min. These bio-chips (called Wab/PEG-SiNWFETs in sample 3 and Fab/PEG-SiNWFETs in sample 4 of Figure 2C) were finally purified by DI-water and desiccated by nitrogen to prepare for the biosensing performance. The manufacturing processes of two-type FET immunosensors by modifying SiNW surfaces with mSAMs of silane-PEG are illustrated in Figure 2.

### 2.4. Biosensing Performance

**Figure 3:**
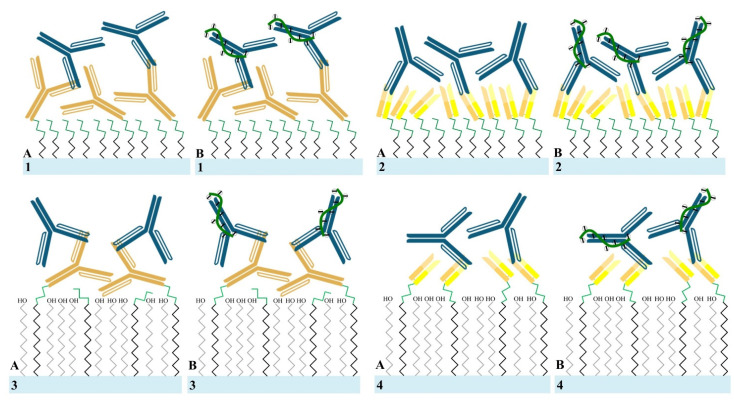
Illustration of the detection of rabbit IgG by (1) Wab/APTES-SiNWFETs, (2) Fab/APTES-SiNWFETs, (3) Wab/PEG-SiNWFETs, and (4) Fab/PEG-SiNWFETs as well as their corresponding signal enhancement by R18 aptamer in this study. Both of the Wab/APTES-SiNWFETs (sample 1) and Fab/APTES-SiNWFETs (sample 2) were used to detect (**A**) rabbit IgG (blue Y shapes) at concentrations of 100 pg/mL and 1 ng/mL, before binding with (**B**) 3 μg/mL R18 aptamer (green curves) for signal enhancement. The Wab/PEG-SiNWFETs (sample 3) could only determine (**A**) rabbit IgG (blue Y shapes) at concentrations of 10 pg/mL, 100 pg/mL and 1 ng/mL, whereas the Fab/PEG-SiNWFETs (sample 4) could recognize (**A**) rabbit IgG (blue Y shapes) at concentrations of 1 pg/mL, 10 pg/mL, 100 pg/mL, and 1 ng/mL. Both of them were then also incubated in (**B**) 3 μg/mL R18 aptamer (green curves) for signal enhancement. All the biosensing experiments in this Figure were performed in 150 mM BTP.

Herein, the detection of rabbit IgG at various levels in 150 mM BTP by the as-prepared sensors and signal enhancement by R18 was implemented following the schematic diagram in Figure 3. To this end, all four types of sensor manufactured in Section 2.3 were initially set-up into the measurement system to record the electrical signal in 150 mM BTP for construction of the first drain current-gate voltage (I_d_-V_g_) curve (the first curve). They were then used to detect IgG at different concentrations in 150 mM BTP for 30 min and washed by DI-water to remove unbound IgG. Subsequently, they were incubated with R18 in 30 min for signal stabilization and amplification and washed by DI-water remove unbinding R18. Eventually, the electrical response after incubation in R18 was recorded at 150 mM BTP, and the second drain current–gate voltage (I_d_-V_g_) curve was built to calculate the electrical variation and analyze the data. All the IgG and R18 solutions used in these experiments were diluted at 1 and 6 μg/mL in 150 mM BTP, respectively. All the electrical measurements were performed in 150 mM BTP, a high-ionic-strength environment equivalent to physiological conditions. The detailed steps of the biosensing performance by each of the four types of SiNWFET immunosensor are illustrated in Figure 3.

### 2.5. Indirect Enzyme-Linked Immunosorbent Assays (ELISA)

**Figure 4:**
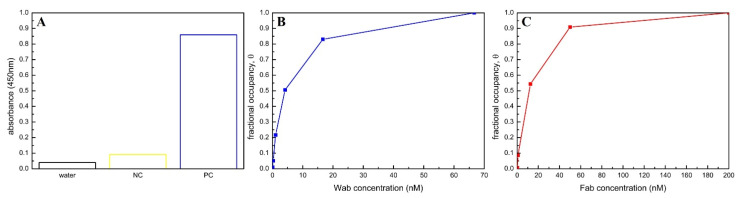
(**A**) Verification of immobilization method with PEG-mSAMs and target-probe binding by indirect ELISA on silica surfaces. Absorbance at 450 nm of pure water (black bar), silica sample modified with PEG-SAMs and GA but without immobilizing IgG (negative control (NC), yellow bar), silica sample prepared with IgG immobilization after modifying PEG-SAMs and GA (blue bar). (**B**,**C**) Plot of the concentrations (nM) of either Wab or Fab bind to IgG versus their corresponding fractional occupancy to determine affinity binding of IgG-Wab (blue curve in (**B**)) and IgG-Fab (red curve in (**C**)).

In order to verify the surface modification, probe immobilization, and the probe-target binding, the IgG were immobilized onto a silica surface modified with PEG-mSAMs to detect the Wab before capturing the donkey anti-goat antibody conjugated with HRP. The silica surface was then reacted with TMB for 30 min and the absorbance was measured at 450 nm. The whole process was also applied for a modified silica surface without IgG immobilization for comparison. The data of this experiment are presented in Figure 4A.

In order to identify the binding between the probe and the target, the IgG were immobilized into an ELISA well to detect either the Wab or the Fab before capturing the donkey anti-goat antibody conjugated with HRP and treated with TMB substrate to measure the light absorbance at 450 nm. In more detail, the rabbit IgG (1 µg/mL) was immobilized onto the ELISA wells and incubated at 4 °C overnight, followed by washing (three times with PBST and one time with PBS) and treating them with blocking buffer (for 2 h before washing again) to eliminate and prevent non-specific adsorption. Subsequently, the wells were filled with either Wab or Fab solution in 30 min and washed with four steps. This process was repeated with HRP-conjugated anti-goat antibody. TMB substrate was then added to the wells for 30 min. Finally, the absorbance of each well was measured at 450 nm after adding HCl solution to stop the process. The whole process was repeated with varied concentrations of the probe (either Wab or Fab) to build the curves displayed relationship between this quantity and their corresponding fractional occupancy (Figure 4B,C), which are used for identifying the binding affinity between the probes (Wab and Fab) and the target (IgG).

### 2.6. Data Analysis of Biosensing by the Manufactured SiNWFET Immunosensors

**Figure 5:**
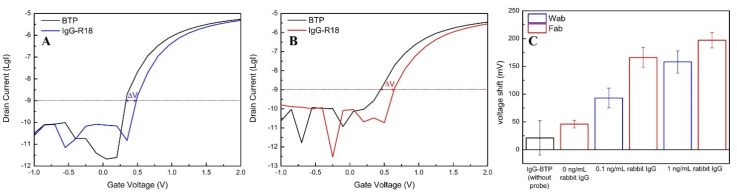
(**A**,**B**) Representative samples to illustrate the method described in Section 2.6. (**A**) Electrical response of the Wab/APTES-SiNWFETs was initially recorded in 150 mM BTP and plotted as the first curve (the black curve). This immunosensor was then employed to detect rabbit IgG at 1 ng/mL, followed by incubation with 3 μg/mL R18 (IgG and R18 were diluted in 150 mM BTP). Finally, its electrical response was measured again and plotted as the blue curve. The signal change generated by formation of the biocomplex (Wab-IgG-R18) was calculated from the formula ΔV = V_d1_ − V_d0_ (1), with V_d1_ as the gate voltage value at I_d_ = 10^−9^ A (LgI = −9) of the blue curve, whereas V_d0_ is the gate voltage value at I_d_ = 10^−9^ A (LgI = −9) of the black curve. (**B**) Electrical response of the Fab/APTES-SiNWFET was initially recorded in 150 mM BTP and plotted as the first curve (the black curve). This immunosensor was then employed to detect rabbit IgG at 1 ng/mL following by incubation with 3 μg/mL R18 (IgG and R18 were all diluted in 150 mM BTP). Finally, its electrical response was measured again and plotted as the blue curve. The signal change generated by formation of the biocomplex (Fab-IgG-R18) was calculated from the formula ΔV = V_d1_ − V_d0_ (1), with V_d1_ is the gate voltage value at I_d_ = 10^−9^ A (LgI = −9) of the blue curve, whereas V_d0_ is the gate voltage value at I_d_ = 10^−9^ A (LgI = −9) of the black curve. (**C**) Comparison of the signal amplified by R18 (mV) after determining rabbit IgG at different concentrations (0.1 ng/mL and 1 ng/mL) in 150 mM BTP by Wab/APTES-SiNWFETs (blue bars) and Fab/APTES-SiNWFETs (red bars). The voltage shift (mV) generated by IgG detection of APTES-SiNWFETs without probes (Wab nor Fab) (black bar), and by recognizing R18 without IgG (0 ng/mL) of Fab/APTES-SiNWFETs, was also calculated for analysis.

Figure 5A,B plotted the method used in this manuscript to analyze the data generated by the as-prepared SiNWFET immunosensors as representative samples. The electrical signal collected before detecting rabbit IgG was constructed as the first I_d_-V_g_ curve (black curves in Figure 5A,B) whereas the electrical signal recorded after incubation with R18 was constructed as the second I_d_-V_g_ curve (blue curve in Figure 5A and red curve in Figure 5B). The signal change before and after the formation of the bio-complex (Wab-IgG-R18 and Fab-IgG-R18) was calculated from the Formula

**Figure 6:**
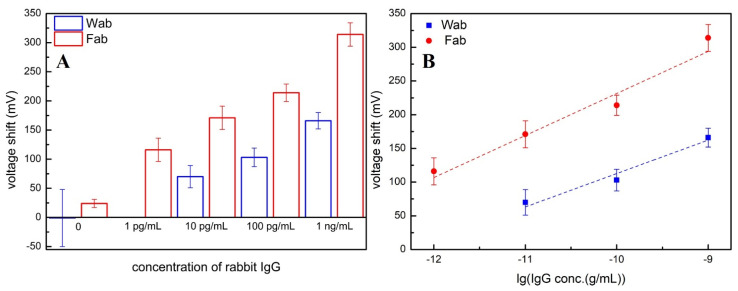
(**A**) Comparison of the signal amplified by R18 (mV) after sensing rabbit IgG at various levels in 150 mM BTP by Wab/PEG-SiNWFETs (blue bars) and Fab/APTES-SiNWFETs (red bars). (**B**) Plot of the voltage shift by R18 versus logarithmic concentrations of rabbit IgG and two respective calibration lines obtained by Wab/PEG-SiNWFETs (blue line) and Fab/APTES-SiNWFETs (red line).

ΔV = V_d1_ − V_d0_ (1)

with V_d1_ as the gate voltage value at I_d_ = 10^−9^ A (LgI = −9) derived from the second curves and V_d0_ as the gate voltage value I_d_ = 10^−9^ A (LgI = −9) derived from the first curves. The statistical data of each experiments (Figure 5C and Figure 6) were collected from three independent devices (n = 3).

## 3. Results and Discussions

### 3.1. Binding Affinity between Probes and Targets by Indirect ELISA and Langmuir Adsorption Model

The absorbance (450 nm) of three samples (water, silica surfaces modified with (PC) or without probes (NC) after detecting the targets and reacting with TMB substrate) is compared in Figure 4A. Obviously, the PC samples (blue column) which underwent probe immobilization present a mostly full absorbance (roughly 90%) in comparison with the trivial values (less than 10%) of pure water (black column) and NC samples (yellow column), indicating that the probes were successfully immobilized onto the SiNW surfaces modified with PEG-mSAMs. Binding affinity (association constant, *K_a_*) of Wab-IgG and Fab-IgG are calculated from data obtained in Figure 4B,C and the Langmuir adsorption model

(2) $$\theta =\frac{K_{a}\times C}{1+K_{a}\times C},$$

where *θ* and *C* represent the fractional occupancy and concentration of either Wab or Fab, respectively. At *θ* = 0.5, Equation (2) becomes

$$0.5=\frac{K_{a}\times C_{0.5}}{1+K_{a}\times C_{0.5}}=\frac{C_{0.5}}{K_{d}+C_{0.5}}\Rightarrow K_{d}=C_{0.5}$$

(*K_d_* is the dissociation constant between the probes (Wab or Fab) and the target (rabbit IgG), *K_d_* = *K_a_*^−1^). Therefore, for Wab-IgG: *K_d_* = *C*_Wab,0.5_ ≈ 4.1 × 10^−9^ M and *K_a_* = 2.4 × 10^8^ M^−1^ (Figure 4B), for Fab-IgG: *K_d_* = *C*_Fab,0.5_ ≈ 1.14 × 10^−8^ M and *K_a_* = 8.8 × 10^7^ M^−1^ (Figure 4C). Obviously, these two *K_d_* figures are within the typical range of *K_d_* values of antibody-antigen binding (10^−6^–10^−9^). On the one hand, this demonstrates that the electrical variations recorded by the FET system are induced from specific binding between the probes (Wab or Fab) and the targets (IgG-R18). On the other hand, the equivalent *K_a_* values in Figure 4B,C indicate that the binding amounts of the targets captured by the probes (Wab and Fab) of both Wab-SiNWFET and Fab-SiNWFETs immunosensors were insignificantly different.

### 3.2. Fab as Bio-Receptors to Improve Amplification Effect of Aptamer for Protein Detection by SiNWFETs Immunosensors

Since protein detection by FETs produced inconsistent trends of signal change [17], it is unable to recognize the antibody-antigen binding via the electrical variation recorded by the FET transducers. Aptamer not only can stabilize, but also significantly amplify the signal change from protein detection by SiNWFETs. Therefore, in this study, we use the voltage shift induced by aptamer to evaluate and analyze the IgG detection by Wab-SiNWFETs and Fab-SiNWFETs. The initial experiments were implemented on these two kinds of sensor, modified with APTES. The voltage shift values enhanced by the aptamer after IgG capture by Wab and Fab probes are calculated by Equation (1) and depicted in Figure 5A,B. Apparently, the voltage shift actuated by Fab/APTES-SiNWFET immunosensors after incubating them with R18 is higher than the similar figures of Wab/APTES-SiNWFET immunosensors. On one hand, the compact structure of Fab, in comparison with Wab, allows these bio-receptors to be immobilized onto the NW surface with a higher density than that of the Wab and allows the Fab/APTES-SiNWFETs to capture more IgG-R18 targets than Wab/APTES-SiNWFETs. On the other hand, the small size of Fab also shortens the distance between negatively charged groups of aptamer and the sensing surface to substantially impact the charge carriers inside the NWs. Consequently, Fab/APTES-SiNWFETs produce greater signal changes after recognizing IgG-R18. This trend is repeatable by the fabricated sensors determining rabbit IgG at two different concentrations of 100 pg/mL and 1 ng/mL (Figure 5C). The electrical variation of the Fab/APTES-SiNWFETs, exposed to the aptamer solution without detecting rabbit IgG (the first red bar to the left of Figure 5C), and the SiNWFETs modified with APTES and GA (without immobilizing Wab nor Fab), incubated with IgG (the black bar in Figure 5C), was collected and calculated to investigate the effect of non-specific binding between the NW surface and the IgG or R18 to the amplified signal. Both resulted in a trivial voltage shift compared to the numbers produced by these two sensors detecting IgG at 100 pg/mL and 1 ng/mL, suggesting an insignificant contribution of nonspecific binding to the experimental data. Therefore, it is feasible to conclude that aptamer as a the signal amplifier for protein detection by SiNWFET immunosensors exhibits an improved performance, with the sensors using Fab as bio-receptors. However, both of the immunosensors manufactured by modifying SiNW surfaces with APTES could only record the amplified signal after detecting the lowest concentration of rabbit IgG at 100 pg/mL, a limitation of high-ionic-strength in the 150 mM BTP solution, which possibly weakened the detected signal and minimized the amplification by R18.

### 3.3. Optimized Protein Quantification by Fab/PEG-SiNWFET Immunosensors and Aptamer Amplifier

After proving the concept of “signal enhancement of aptamer for protein detection by FETs is improved with Fab as bio-receptors” on the APTES-SiNWFET immunosensors, we further examine it for PEG-SiNWFET immunosensors. PEG not only prevents the non-specific adsorption of rabbit IgG and R18 on the NW surface to minimize their contribution to the signal change in the immunoassay enhanced by the R18, but also extends the small Debye length in a high-ionic-strength environment and correspondingly maximize the electrical variation recorded by the FET transducers. Figure 6A characterizes and compares the voltage shift produced by Wab/PEG-SiNWFETs and Fab/PEG-SiNWFETs after detecting rabbit IgG (at various levels in 150 mM BTP) and R18. Similar to the results obtained in the trials with APTES-SiNWFET immunosensors, the signal change recorded by Fab/PEG-SiNWFETs after capturing R18 was always greater than that of Wab/PEG-SiNWFETs (almost double), although both types of sensors detected the same concentrations of rabbit IgG (10 pg/mL, 100 pg/mL, 1 ng/mL). Moreover, the Fab/PEG-SiNWFET immunosensors achieved the detection limit to determine the IgG level down to 1 pg/mL in the range of 1 pg/mL–1 ng/mL (ΔV_R18-BTP_ = (62.3 ± 10)lgC_Aβ1-42_ + (854.9 ± 105), R = 0.93), whereas the Wab/PEG-SiNWFET immunosensors can only detect the smallest concentration of IgG at 10 pg/mL in the range of 10 pg/mL–1 ng/mL (ΔV_R18-BTP_ = (49.6 ± 8.8)lgC_Aβ1-42_ + (608.4 ± 86.2), R = 0.94) (Figure 6B). A comparison between two calibration lines also reveals the superior sensitivity of Fab/PEG-SiNWFETs. Similar to the reasons explained in the trials with APTES-SiNWFET immunosensors, the advantage in terms of the dimension of the Fab probes, which is increasingly favorable under the effect of enlarged Debye length by PEG, plays a major role in the impressive performance of Fab/PEG-SiNWFETs in comparison with Wab/PEG-SiNWFETs. Furthermore, PEG chains along the mSAMs structure also form a hydration layer to avoid the nonspecific adsorption of R18 onto the modified surface, minimizing its contribution to the signal changed by specific binding (Figure 6A). Apparently, voltage, shifted by the non-specific binding of aptamer to the modified surface, was remarkably decreased in Fab/PEG-SiNWFETs, compared to the value from Fab/APTES-SiNWFETs. A detailed comparison between signals changed by aptamer at the same concentrations (100 pg/mL and 1 ng/mL) reveals that the one obtained by Wab/PEG-SiNWFETs is slightly higher than that of Wab/APTES-SiNWFETs. In addition to expanding the detectable region of FET and anti-fouling function, the spacer effect of PEG also prevented the bio-receptors (Wab and Fab) from directly contacting with the Si channels [25,26]. Consequently, their bioactivities are retained to contribute to the sensitivity of PEG-SiNWFETs. This trend is much more obvious in the comparison between the voltage shift by Fab/PEG-SiNWFETs and Fab/APTES-SiNWFETs, suggesting that the combination between aptamer as the signal amplifier and Fab as probes for SiNWFET immunosensors is optimized under the SiNWFETs modified with PEG. More importantly, the *K_d_* and *K_a_* values calculated from the data in Figure 4B,C not only, on the one hand, indicate that the electrical variations obtained from this experiment are induced from specific binding between the probes (Wab or Fab) and the targets (IgG-R18), but also, on the other hand, demonstrate that the greater signal change in Fab-SiNWFETs was due to the reduced size of Fab probes, since the amount of targets captured by both Fab-SiNWFETs and Wab-SiNWFETs were analogous because of their equivalent *K_a_* values. In comparison with previous publications using Fab as the bio-recognition factor for immunosensors based on FETs [10,13,19,20,21,22], this is the first study not only combining both Fab and aptamer for the synergic signal enhancement of immunoassays by FETs, but also experimentally proving that the remarkable amplification effect came from the compact structure of the Fab bio-receptors.

## 4. Conclusions

Determining proteins at low concentrations in high-ionic-strength conditions by SiNWFETs is limited by the small Debye length and deteriorated signal. Herein, a method for this problem integrating aptamer as signal amplifier into SiNWFET immunosensors and employing Fab as bio-receptor is presented for the first time. The comparison with SiNWFETs using Wab indicates that the enhanced signal was significantly improved by the SiNWFETs with Fab probes. Moreover, the amplified signal by aptamer is optimized in combination with SiNWFETs modified by PEG and Fab, in which the surface modification method and probe-target binding were verified by indirect ELISA to recognize the target down to 1 pg/mL in high-ionic-strength solution. Empirical results were given by indirect ELISA and the Langmuir adsorption model, which, for the first time, proved that (1) the electrical variations were generated by specific binding between the probes (Wab or Fab) and the target (IgG-R18), and (2) the synergic signal enhancement of combination between Fab and aptamer were from the compact structure of the Fab. The proposed strategy, therefore, has potential for further applications to detect proteins in high-ionic-strength conditions by SiNWFETs.

## Data Availability

Data sharing not applicable.
